# Challenge or Hindrance? The Dual Path Effect of Perceived Task Demand on In-Role Performance and Work Fatigue

**DOI:** 10.3390/ijerph192315561

**Published:** 2022-11-23

**Authors:** Zhigang Li, Xin Zhang, Junwei Zheng, Zhenduo Zhang, Pengyu Wan

**Affiliations:** 1 School of Economics and Management, Beijing Polytechnic, Beijing 100176, China; 2 Graduate School of Education, Dalian University of Technology, Dalian 116024, China; 3 Faculty of Civil Engineering and Mechanics, Kunming University of Science and Technology, Kunming 650500, China; 4 School of Economics and Management, Dalian University of Technology, Dalian 116024, China; 5 School of Economics and Management, Chongqing Normal University, Chongqing 401331, China

**Keywords:** perceived task demand, cognitive engagement, cognitive stain, in-role performance, work fatigue

## Abstract

The evidence for the existence of perceived task demand is paradoxical. The purpose of the present study is to explore whether perceived task demand is a challenge or a hindrance stressor. To achieve this research purpose, based on conservation of resources theory, a conceptual model is developed that utilizes both a resource acquisition path and a resource depletion path. Using the experience-sampling method, over five consecutive days, 370 matched data were collected via mobile phone from 74 full-time employees in mainland China. The results show that perceived task demand has the characteristics of both challenge and hindrance stressors. On the one hand, perceived task demand enhances employees’ cognitive engagement, thereby facilitating task performance (resource acquisition path). On the other hand, perceived task demand boosts employees’ cognitive strain, thereby increasing work fatigue and decreasing in-role performance (resource depletion path). This research offers a comprehensive understanding of perceived task demand and provides strategies for task demand management.

## 1. Introduction

Job demands are the physical, psychological, organizational, and social aspects of a job that require sustained investments of personal, physical, and/or psychological resources (skills and efforts) [1]. Job demands have long been regarded as being detrimental to employees’ wellbeing. For example, Schaufeli [2] found that job demands increased employee burnout and decreased employability.

Based on previous research [3], Bakker and Sanz-Vergel [4] divided job demands into two different categories: challenge and hindrance demands. According to this definition, challenge demands are obstacles that can be overcome, thus facilitating employees’ learning and achievements within organizations [5]. Challenge demands provide the benefits of stimulating employees’ intrinsic motivation and enhancing their in-role performance [3,6,7]. Hindrance demands contrast with challenge demands, denoting those unnecessary demands that hinder personal growth and goal attainment [5]. By this definition, hindrance demands impose direct negative impacts on employees’ growth at work [8,9].

Perceived task demand is a special kind of job demand that arises in situations where a substantial amount of work must be completed under great time pressure [10]. Perceived task demand used to be examined as a hindrance demand associated with role stress, increased work fatigue, and work–family conflict [10]. However, research has also identified beneficial aspects of perceived task demand. For example, Garcia et al. [11] and Schyns et al. [12] explored the positive relationship between perceived task demand and job satisfaction. Considering the transformation of the economic system and the high-speed development of China, Chinese employees are more likely than ever to face increased task demands [13,14,15]. In particular, after the outbreak of the COVID-19 pandemic, nearly 74% of female Chinese employees have reported that their task demands have increased [16]. Thus, the purpose of the present research is to explore—in detail—the characteristics of the perceived task demand and assess whether it is a challenge or a hindrance stressor.

Given that perceived task demand is a mutual concept with clear definitions and methods of measurement, the changes in personal resources stimulated by perceived task demands were adopted to obtain insights into the challenge and/or hindrance aspects of demands such as those proposed by Breevaart and Bakker [17]. To this purpose, in this paper, a multilevel model is developed within the framework of the conservation of resources (COR) theory. COR theory provides a comparative perspective for exploring the challenge and hindrance characteristics of perceived task demand [18]. It achieves this by proposing that individuals invest personal resources to cope with challenge demands and gain resources while attempting to minimize the loss of personal resources. Initial losses and gains of resources result in future resource losses and gains, respectively [19]. Previous research has highlighted the influences of challenge demands on personal resource gains [13] and of hindrance demands on resource losses [20]. In line with these assumptions, in this paper, two divergent paths (resource acquisition and resource depletion paths) for perceived task demand are developed. From the perspective of resource acquisition, perceived task demand enhances employees’ cognitive engagement, thereby facilitating in-role performance and attenuating work fatigue. From the perspective of resource depletion, perceived task demand increases cognitive strain, leading to increased work fatigue and decreased in-role performance.

The reason why in-role performance and work fatigue were chosen as indicators of perceived task demands is that management researchers are focusing on the effectiveness and subjective well-being of their employees [14]. In-role performance comprises the required outcomes and employee behaviors described in the employees’ job profiles, which contribute to the goals of the organization [21]. Maintaining high-level in-role performance is the distal goal of all companies’ policies and strategies. Studies have proved that sufficient psychological resources are essential for high-level in-role performance [22]. By contrast, studies have suggested that work fatigue is widespread in organizations and undermines organizational effectiveness [23]. Work fatigue reflects the feelings of fatigue a worker experiences following exposure to job demands during a work shift. These can be characterized by states of physical exhaustion and temporary impoverishment in cognitive function, mood, and motivation [24]. In contrast to in-role performance, the availability of sufficient resources would effectively avoid the emergence of work fatigue [25]. Considering the correlations between changes in resources and in-role performance and work fatigue, this study adopts in-role performance and work fatigue as the two outcomes of perceived task demands based on the theoretical lens of the COR theory. This approach provides a comprehensive insight into the outcomes of perceived task demand in organizations.

The experience-sampling method was adopted to test the dual-path model on a daily basis. Thus, this research contributes twofold to the job demands literature. First, empirical evidence is provided for the hindrance characteristic of perceived task demand and the constant depletion of personal resources leading to cognitive strain and work fatigue. Second, this research further explores the challenge characteristic of perceived task demand. This challenge can motivate employees to acquire personal resources, resulting in increased cognitive engagement and in-role performance.

## 2. Hypothesis Development and Literature Review

### 2.1. Resource Acquisition Path of Perceived Task Demand

The previous research identified work engagement and in-role performance as two of the most important work-related outcomes caused by challenge demands from the perspective of resource acquisition [4,17,26,27]. To explore the challenge characteristic of perceived task demand, the present paper develops a resource acquisition path, which starts from perceived task demand, progresses to cognitive engagement, and advances to in-role performance and work fatigue. Rather than other dimensions, this research examines cognitive engagement as a crucial mediator, which is defined as a psychological presence and focus at work. Kahn [28] divided work engagement into the three dimensions of emotional engagement, physical engagement, and cognitive engagement. Kahn [28] noted that people exhibited engagement when they became physically involved in tasks, whether alone or with others (i.e., physical engagement), when they were emotionally connected to their work and to others in the service of their work (i.e., emotional engagement), and when they were cognitively vigilant, focused, and attentive (i.e., cognitive engagement).

Cognitive engagement is interpreted through multiple lenses of work, including those of meaningfulness, safety, and resources [28]. Employees showing cognitive engagement tend to be mentally vigilant, focused, and absorbed in their work [29]. Cognitive engagement has been identified as an important resource for employees to maintain job performance and ongoing career development [30]. Moreover, it is important to note that the three dimensions of work engagement (i.e., cognitive, emotional, and behavioral) are interrelated [31]. Prior research pointed out that changes in emotional and physical resources are the conclusions of changes in cognitive resources [32]. This suggests that changes in cognitive resources are more proximal outcomes of perceived task demands. Considering the importance of cognitive engagement and sensitivity to changes in perceived task demands, this work explicitly explores the phenomenon of cognitive engagement as a potential mediator in the relationship between perceived task demands and both in-role performance and work fatigue.

Studies based on COR theory have proposed that challenge stressors act as a beneficially stressful context that motivates individuals to acquire resources through overcoming difficulties and completing task demands [33]. Consistent with prior studies, the present paper suggests that perceived task demand activates positive outcome expectancy. This, in turn, stimulates an increase in employees’ cognitive engagement through the belief that meeting task demands can provide opportunities for personal gains. Task demands attribute importance and meaningfulness to tasks, thus stimulating employees to exert extra efforts to meet task demands [34,35]. This suggests that perceived task demand is a signal to employees that an opportunity for personal gain exists, thereby representing important events that affect employees’ self-worth and work lives [12]. A high level of perceived task demand increases the probability of obtaining salary incentives and job promotions [36], both of which increase the levels of cognitive engagement [17].

In addition, COR theory is a motivation theory in nature, suggesting that individuals are motivated to acquire resources through beneficial interactions with beneficial contexts. Perceived task demand is associated with a high level of task complexity and autonomy [12], which requires employees to direct their cognitive resources toward the completion of the tasks at hand [37,38]. Investing resources into meeting these task demands can facilitate employees’ pursuit of opportunities for future rewards [3]. Employees with high task demands will, therefore, exert increased efforts and cognitive engagement towards job tasks [39]. Thus, the following hypothesis is proposed:

**Hypothesis** **1** **(H1).** *Daily perceived task demand positively relates to daily cognitive engagement*.

Cognitive engagement is a state in which employees are focused on their work and are less easily distracted from their work by things peripheral to their jobs and by unrelated problems arising in the course of work [40]. The cognitive engagement literature suggests that cognitively engaged employees are better able to overcome work-related obstacles and are more effective at achieving work-related goals, thereby facilitating their in-role performance [41].

COR theory proposes that employees with more job resources are more likely to experience resource gains by investing existing job resources [42]. Cognitive engagement is a means of allotting personal resources so as to enhance cognitive capacity at work [41]. The increased work-related cognitive capacity improves in-role performance because cognitively engaged employees are better at generating ideas and solving work-related problems [43,44]. Furthermore, improving in-role performance requires employees to proactively seek opportunities and adopt effective actions toward self-development in an effort to meet increasing job requirements [45]. Ho et al. [41] proposed that employees with high levels of cognitive engagement are better prepared to exhibit proactive behaviors at work, thereby increasing their in-role performance.

In addition to promoting in-role performance, the present paper also assumes that cognitive engagement is negatively associated with work fatigue. COR theory suggests that the availability of sufficient resources is vital for individuals to maintain their well-being and cope with fatigue at work [46]. Prior studies provided evidence for the benefits of cognitive engagement. For instance, Sonnentage et al. [47] found that engagement is positively related to employees’ recovery experiences and negatively related to fatigue experiences at a daily level. Cognitively engaged employees easily focus on the task at hand and exhibit goal-directed behavior [48]. Thus, cognitive engagement facilitates employees to accomplish their work tasks without having to frequently mobilize their attentional or additional self-regulatory resources, which would lead to work fatigue [47]. In other words, when employees are cognitively engaged at work, only a few extra cognitive resources must be consumed or mobilized to finish work tasks. Consequently, cognitive resources will still be available at the end of the workday, and work fatigue can be impeded because of the conservation of cognitive resources [49]. Thus, the following hypothesis is proposed:

**Hypothesis** **2** **(H2).** *Daily cognitive engagement positively relates to daily in-role performance (+, a) and negatively relates to daily work-fatigue (−, b)*.

Perceived task demands represent job demands that are associated with task complexity and autonomy, and carry the potential to increase mastery, personal growth, or future gains [34]. Employees are likely to exert significant effort towards finishing perceived task demands on time to attain rewards and achieve personal growth [50]. As such, it is reasonable to argue that employees who experience perceived task demands pay more attention to their work and possess higher levels of cognitive engagement in their jobs, thereby enhancing in-role performance and decreasing work fatigue. Accordingly, the following hypothesis is proposed:

**Hypothesis** **3** **(H3).** *The relationships between daily perceived task demand and daily in-role performance (+, a) and daily work fatigue (−, b) are mediated by daily cognitive engagement*.

### 2.2. Resource Depletion Path of Perceived Task Demand

Job strain and work fatigue have been identified as important work-related consequences of hindrance demands [51,52]. Job demands are those aspects of a job that are constantly depleting personal resources [1]. COR theory assumes that the sustained loss of job resources will lead to job strain and its associated work behavior [19].

Psychological strain is a state of mental impairment resulting from a perceived goal-discrepancy [53]. Cognitive strain is the cognitive aspect of psychological strain, denoting a class of conscious thought concerning a common instrumental theme that recurs even in the absence of immediate environmental demands requiring such thoughts [53]. In the organizational context, cognitive strain refers to a rumination state in which employees frequently think about work-related affairs involving automatic and controlled processing, which obstructs goal attainment [53]. As mentioned above, changes in cognitive resources are antecedent to changes in emotional resources [32]. To explore how perceived task demands stimulate resource depletion, this research adopts cognitive strain as the proximal outcome of perceived task demands.

Resources are important for individuals to be able to cope with job demands. When job resources are insufficient, employees are more likely to experience negative work-related rumination and cannot distract themselves from their work [42]. Cognitive strain derives from stressful life experiences [54,55] and results in a slow recovery from stressful work experiences [56].

High task demands entail large amounts of work that must be completed, where employees must work particularly hard and fast under time pressure [10]. Previous research has indicated that individuals tend to recall negative work events as a maladaptive response to high task demands [57]. Employees who are experiencing high task demands will not easily disengage from work-related matters because of their high-level engagement in a previous task to meet certain job requirements [42]. Prior research also provided evidence for the positive relationship between perceived task demand and cognitive strain. Cropley and Purvis [57] found that when teachers were experiencing significant job strain, they tended to report a high volume of daily work-related ruminative thoughts. In addition, employees found it difficult to switch off after work when faced with high amounts of work stressors. Taken together, the following hypothesis is proposed:

**Hypothesis** **4** **(H4).** *Daily perceived task demand positively relates to daily cognitive strain*.

Work fatigue refers to a reduced psychological capacity and a reduced willingness to perform adequately because of mental or physical efforts exerted earlier [58]. Empirical evidence has been provided for the existence of a relationship between cognitive strain and work fatigue [59,60,61,62]. The core construct of cognitive strain is the inability to switch off after work [53]. Cognitive strain, or work rumination, is associated with increased basal sympathetic activation, which eventually leads to vital exhaustion.

Cognitive strain leads to the prolonged activation of the cardiovascular, endocrinological, immunological, and neurovisceral systems [59,63]. The inability to withdraw from work obligations involves experiences of repetitive, negative, and intrusive cognition associated with depression over past work-related affairs [64]. Thus, cognitive strain inhibits employees’ recovery from work [65]. The lack of detachment from a previous workday has been identified as a critical antecedent of work fatigue and negative activation on the following day [66]. Further, research has provided evidence for the existence of a relationship between cognitive strain and poor sleep quality, which further inhibits employees’ recovery from work and promotes work fatigue.

In addition, consistent resource depletion threatens both the mental health and in-role performance of employees. The engendered cognitive strain prolongs work-related activation and prevents employees from adequately recovering from job demands [47]. From a resource depletion perspective, this impaired recovery experience engenders lower levels of in-role performance at work. The underlying reasoning is that insufficient recovery experiences prevent the replenishment of cognitive resources. This, in turn, leads employees to prioritize the devotion of their limited resources to deviant workplace behavior so as to conserve and obtain resources at the expense of their in-role performance. Taken together, COR theory suggests that the experiences of job strain consistently consume personal resources and, consequently, lead to enhanced fatigue and decreased in-role performance. Thus, the following hypothesis is proposed:

**Hypothesis** **5** **(H5).** *Daily cognitive strain positively relates to daily work fatigue (+, a) and negatively relates to daily in-role performance (−, b)*.

**Figure 1:**
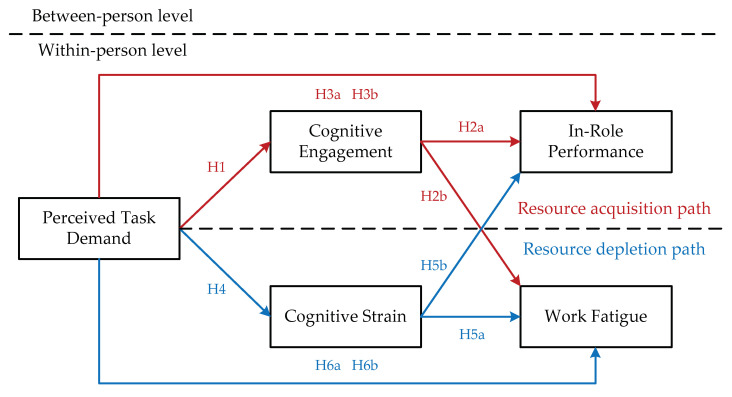
The dual-path model.

Perceived task demand derives from a large number of tasks that must be competed under time pressure, which constantly depletes personal job resources and leads to employees’ cognitive strain. Cognitive strain involves repetitive and instructive cognition regarding work-related affairs, thereby preventing employees from recovering from work and leading to both work fatigue and undermined in-role performance. Hence, the following hypothesis is proposed, and Figure 1 depicts the proposed model:

**Hypothesis** **6** **(H6).** *The relationships between daily perceived task demand and daily work fatigue (+, a) and daily in-role performance (−, b) are mediated by daily cognitive strain*.

## 3. Methods

### 3.1. Sample

Researchers adopt the experience-sampling method to study human behavior. Respondents in experience-sampling studies are asked to provide self-reported activities, emotions, and other elements of their daily life either once or multiple times per day or per week. When sampling employees, their responses capture fluctuations of their work experiences and the proximal relationships between focal variables [67].

For both theoretical and empirical reasons, a multilevel, multiday diary design was utilized. Theoretically, COR theory is a dynamic theory, as resource and demand levels ebb and flow both between and within individuals [68]. Furthermore, this research focused on the proximal consequences of perceived task demands, which necessitates a daily diary design. Therefore, the utilized methodology is appropriate, given the applied theoretical lens and the nature of the focal phenomena discussed in the proposed model. From an empirical standpoint, the use of daily experience-sampling method is justified because perceived task demands vary daily [69].

A subject pool was developed comprising 113 alumni of a management school at a university in Beijing, China. Specific criteria were established to screen for appropriate samples. First, alumni had to have updated their contact information within the past two years. Second, they had to have been full-time workers (over 40 h per week) in mainland China. With the help of the management school secretary, the participants were contacted via email, telephone, and mobile phone social apps (i.e., WeChat and QQ, both mobile phone social apps available in China). The purpose of the research and the five-day research procedure were explained to the participants. Finally, 76 alumni confirmed their participation in the survey.

A two-wave experience-sampling method design was adopted, and a group was formed in WeChat. The participants were asked to finish the questionnaire on a Sunday (including gender, education, age, and ID). Then, on the following weekdays (Monday to Friday), they were required to finish one questionnaire at 11:00 (assessing perceived task demand) and another at 19:00 (assessing cognitive engagement, in-role performance, cognitive strain, and work fatigue). Since two participants failed to finish the initial survey, 370 matched data were finally collected, which were nested in 74 samples over five consecutive days. The effective response rate was 97.4%.

Participants were mainly employed in manufacturing and electronic industries. Males accounted for 44.6% of the participants, participants holding college certifications or below accounted for 6.8%, and participants holding bachelor’s degrees accounted for 68.9% of the samples. The average age of participants was 29.08 (±4.76) years.

### 3.2. Measure

All scales adopted in this study originate from top peer-reviewed journals and were originally published in English. A standard back-translation procedure was employed to translate the scales into Chinese. A five-point Likert scale was adopted in this study, where 1 indicated “strongly disagree” and 5 indicated “strongly agree”. It should be mentioned that because the experience-sampling method was used in this study, respondents were required to repeatedly complete the same questionnaires. To ensure the response rate, short-form questionnaires were adopted by selecting the items with the highest loadings in the original questionnaires [70,71]. Before the questionnaire survey was conducted, a preliminary cross-sectional study was conducted to ensure that the short-form questionnaire achieved acceptable validity. The results of correlation analysis indicated that the short-form questionnaires of perceived task demand (*r* = 0.84 and *p* < 0.01), cognitive engagement (*r* = 0.79 and *p* < 0.01), in-role performance (*r* = 0.83 and *p* < 0.01), and cognitive strain (*r* = 0.91 and *p* < 0.01) had significant relationships with the original questionnaires. This result justifies the use of the short-form questionnaires.

Perceived task demand was measured by three items originally developed by Williams and Alliger [10]. A sample item was, “today, I needed to work hard at my work”. The scale yielded an average Cronbach’s alpha of 0.86. Cognitive engagement was measured by two items from the scale originally developed by Kahn [28]. A sample item was, “today, I was rarely distracted when performing my job”. The average reliability coefficient of this scale was 0.81. In-role performance was measured by two items originally developed by Williams and Anderson [72]. A sample item was, “today, I adequately completed assigned duties”. The average Cronbach’s alpha of this scale was 0.88. Cognitive strain was measured by two items originally developed by Mohr et al. [53]. A sample item was, “today, I thought of my problems at work, even at home”. The scale yielded a reliability coefficient of 0.70. Work fatigue was measured by three items originally developed by Michielsen et al. [58]. A sample item was, “today, I was bothered by fatigue”. The Cronbach’s alpha of this scale was 0.83.

Based on previous research, as control variables, gender, education, and age were adopted because of their potential influences on in-role performance and work fatigue [61,73]. However, the parameters in the multilevel structural equation model did not change significantly, regardless of whether these control variables were adopted in the statistical model. Hence, according to suggestions made by Aguinis and Vandenberg [74], a research model without control variables was adopted and the results with control variables were displayed in Appendix A.

## 4. Results

### 4.1. Multilevel Confirmatory Analysis

Considering that daily data are nested in nature, a multilevel confirmatory analysis (MCFA) was adopted to test the validity of the five-factor conceptual construct. At the within-person level, perceived task demand, cognitive engagement, in-role performance, cognitive strain, and work fatigue were included. The hypothesized five-factor conceptual model achieved a better fit for the data (χ^2^(44) = 2.800, RMSEA = 0.07, SRMR = 0.05, CFI = 0.96, and TLI = 0.94) than the baseline one-factor construct (△χ^2^(4) = 28.93 and *p* < 0.01). Table 1 presents the results of the MCFA.

### 4.2. Descriptive Statistics

Table 2 provides the descriptive statistics, intra-correlations, and correlations for the study variables. The results preliminarily verified the hypotheses.

### 4.3. Regression Analysis

In the first stage of the statistical analysis, the within-person variance was systematically studied for episodic variables. The within-person variances for perceived task demand, cognitive engagement, in-role performance, cognitive strain, and work fatigue were 0.44, 0.48, 0.50, 0.39, and 0.47, respectively, corroborating the appropriateness of the multilevel analysis.

The hypotheses were examined via multilevel mediation analysis using the Mplus 7.04 software with a random slope. Before a multilevel structural equation model was run, the within-person variables were all group-centered.

Table 3 shows that perceived task demand was positively correlated with cognitive engagement (*γ* = 0.13; *p* < 0.01), and cognitive engagement was positively correlated with in-role performance (*γ* = 0.35; *p* < 0.01) but not with work fatigue (*γ* = 0.01; *n.s*.). Hypotheses 1 and 2a are supported, but hypothesis 2b is not supported. Further, the direct influence of perceived task demand on in-role performance was not significant (*γ* = −0.03; *n.s.*; 95% Confidence Interval = [−0.09, 0.03]), while the indirect influence through cognitive engagement was significant (*γ* = 0.05; *p* < 0.01; 95% Confidence Interval = [0.02, 0.08]). However, the indirect influence of perceived task demands on work fatigue through cognitive engagement was not significant (*γ* = 0.01; *n.s.*; 95% Confidence Interval = [−0.01, 0.01]). The results of the Monte-Carlo bootstrapping tests conducted in R studio (Version 3.5.3) supported Hypothesis 3a, but not Hypothesis 3b (Table 4).

By contrast, perceived task demand was positively associated with cognitive strain (*γ* = 0.22; *p* < 0.01), and cognitive strain was positively related to work fatigue (*γ* = 0.10; *p* < 0.01) and negatively related to in-role performance (*γ* = −0.17; *p* < 0.01). Further, the direct influence of perceived task demand on work fatigue was significant (*γ* = 0.32; *p* < 0.01; 95% Confidence Interval = [0.24, 0.40]). The indirect influences of perceived task demand on work fatigue through cognitive strain (*γ* = −0.02, *p* < 0.05, and 95% Confidence Interval = [0.01, 0.04]) and on in-role performance (*γ* = −0.04, *p* < 0.05, and 95% Confidence Interval = [−0.06, −0.02]) were significant. Therefore, Hypotheses 4, 5a, 5b, 6a, and 6b are supported.

**Figure 2:**
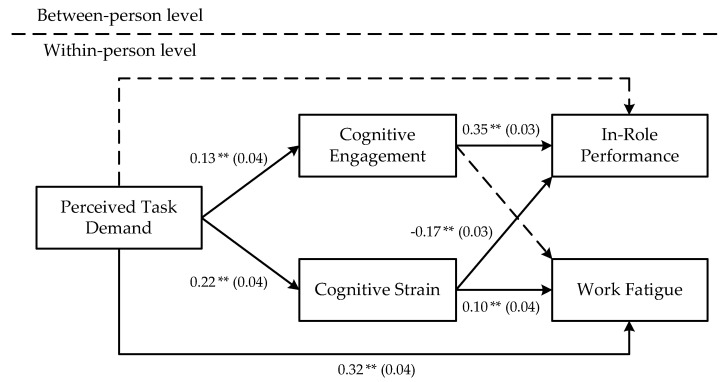
Conceptual model and results of multilevel structural equation modeling. Notes: values in parenthesis are standard errors; parameters are unstandardized; ** *p* < 0.01.

Finally, the conceptual model and results of the multilevel structural equation model were obtained (see Figure 2).

## 5. Discussion

### 5.1. Theoretical Implications

This research provides two theoretical contributions to the perceived task demand literature. First, by disclosing the resource depletion path of perceived task demand, empirical evidence has been provided for the hindrance characteristic of perceived task demand. As a typical job demand, research has explored the association between perceived task demand and job stress, negative mood, and work–family conflict [10,75,76]. COR theory suggests that contextual demands consistently consume personal resources. The result of this study is consistent with this line of research, as it uncovered the depletion of personal resources. Perceived task demand derives from a heavy workload that must be accomplished under time pressure [10]. Moreover, this research contributes to COR theory and perceived task demand by specifying depleted resources as cognitive resources.

Although job demands have resulted in a loss of cognitive, emotional, and physical resources, the loss of cognitive resources tends to be a proximal response to job demands [42]. Faced with high task demands, employees tend to be over-engaged in their work, making it difficult for them to distract themselves from their jobs, even while at home [42]. In this context, employees will experience repetitive and intrusive thinking about their heavy workload, leading to associated anxiety and worry, i.e., cognitive strain [77]. Due to its association with prolonged psychological and biological activation, cognitive strain further prevents employees from recovering from heavy task demands and maintaining high-level task performance [59]. Within COR theory, the present research unveils how the hindrance stressor aspect of perceived task demand hinders employees’ development in organizations through the depletion of cognitive resources. This provides evidence for the potential fluctuations of the hindrance characteristic of perceived task demand at the episodic level.

Second, in this research, the challenge influence of perceived task demand on in-role performance and work fatigue was explored through cognitive engagement, thus deepening the insights into the benefits of perceived task demand. Through the lens of COR theory, the fundamental difference between challenge and hindrance demands is that a challenge demand stimulates employees’ expectations to realize self-development through overcoming certain challenging obstacles, while a hindrance demand does not provide such stimulation but consistently consumes personal resources [78,79,80]. Previous research argued that perceived task demand is associated with complexity and autonomy, thereby enhancing employees’ motivations to meet task demands and concentrating employees’ attention on their current work [11,12]. This research identifies cognitive engagement as a resource obtained through perceived task demand. The results extend previous studies by clarifying the correlations between perceived task demand and cognitive engagement, work fatigue, and in-role performance.

COR theory proposes that employees are motivated to invest personal resources to achieve resource gains [19]. A high task demand is usually linked to challenges and opportunities, signaling to employees that they could master related skills and acquire career development through overcoming task demands [11,12]. Therefore, to meet high task demands, employees must direct their cognitive resources toward their present jobs and enhance their cognitive capacities [41]. As a result, increased in-role performance is achieved through effective coping strategies and fewer additional efforts are needed to mobilize their attention to non-work-related affairs [81,82]. However, it should be noted that cognitive engagement cannot significantly decrease work fatigue. The reason has been presented in prior studies that have also addressed the negative aspect of excessive cognitive engagement. When cognitive engagement is excessively high, individuals may have fewer opportunities to distract themselves from their work, thereby enhancing the probability of experiencing work fatigue [83]. The present research explored the motivating influences of perceived task demand on employees toward obtaining extra personal resources and deepens insight into the challenge characteristics of perceived task demand.

### 5.2. Practical Implications

The results of this research will help to guide managers toward adopting a holistic view of employees’ perceived task demand. Managers should realize that perceived task demand can be both a motivator to improve in-role performance and a deterrent to work fatigue. Therefore, maximizing the challenging effects of perceived task demand while circumventing their hindering influences must constitute the central orientation of management practice.

First, managers should set reasonable and acceptable work tasks according to the different qualities of their employees. On this basis, employees should be guided to view perceived task demand as an opportunity for self-development and for enhancing their ability and beliefs to cope with task demand. For example, managers should help employees to establish a concept of active adaptation, accept challenges, enhance their ability to solve task demands, and establish a multifaceted social support system that includes family, colleagues, leaders, and professional physicians.

Second, in addition to perceived task demand, managers should simultaneously pay attention to employees’ daily work engagement and job strain. The goal should be to make the perceived task demand feasible, thus optimizing the challenging influences of perceived task demand. On the one hand, managers should implement a fair and functional feedback reward mechanism corresponding to the process of employees completing work tasks, combined with creating a harmonious working atmosphere. Employees should receive more understanding and necessary work resources to complete tasks, thus increasing their work engagement. On the other hand, managers need to channel employees’ job strain in a timely manner, encourage them to engage in time planning and task management, actively participate in sports, and restore their work energy by combining work and rest.

### 5.3. Limitation and Future Research

This research has certain limitations that may indicate directions for future research. First, this research could not establish firm causal relationships between the focal variables of this study. A two-wave experience-sampling method was used to test the conceptual model, which better allows researchers to infer the relationship between perceived task demand and its work-related consequences [84]. However, other variables were collected at the same time, which prevented the further exploration of the relationships among them, especially the relationships between work fatigue and in-role performance (correlation: *r* = 0.25 and *p* < 0.01), and between cognitive engagement and cognitive strain (correlation: *r* = 0.32 and *p* < 0.01). Future research could employ a multi-wave, cross-lagged panel design or an experimental design as these might better establish a convincing causal effect [85,86].

Second, in the present study, common method variance (CMV) could not be ruled out [87]. All data were collected via self-reported questionnaires, introducing the potential bias of CMV. Although the result of the MCFA suggested that the effects of CMV might be minimal, future research could collect data from multiple sources to further decrease CMV [88]. For instance, cognitive engagement and in-role performance could be rated by organization leaders.

Third, this study could not discern the condition under which perceived task demand exhibits more challenge characteristics or hindrance characteristics. This research unveiled the challenge and hindrance paths through which perceived task demand impacts personal psychological states and work behavior. However, the mechanisms through which the hindrance and challenge paths of perceived task demand can be transferred to each other were not further explored. COR theory proposes that contextual and personal resources influence resource acquisition and depletion [19,68]. Future research could consider factors such as positive leadership, job control, and the team climate [89,90,91] as boundary conditions to obtain a more comprehensive insight into perceived task demand.

## 6. Conclusions

In this research, the challenge and hindrance characteristics of perceived task demand were explored at the episodic level. Perceived task demand is both a challenge and a hindrance stressor. Indeed, perceived task demand constantly depletes personal resources, and the sustained depletion of job resources causes increased cognitive strain and work fatigue. However, perceived task demand also provides employees with job development opportunities, thereby motivating them to cognitively engage in their work and to enhance their in-role performance. This research integrates previous research, addresses the different aspects of perceived task demand, and offers a comprehensive understanding of perceived task demand.

## Figures and Tables

**Table 1:** Results of multilevel confirmatory factor analysis.

Model	Variables	χ^2^	ds	△χ^2^	RMSEA	SRMR	CFI	TLI
Model 1	PT, CE, IRP, WF, CS	123.22	44		0.07	0.05	0.96	0.94
Alternative Model								
Model 2	PT, CE, IRP, WF + CS	152.14	48	28.93 **	0.08	0.06	0.94	0.92
Model 3	PT, CE + IRP, WF, CS	306.37	48	183.15 **	0.12	0.08	0.86	0.80
Model 4	PT + CE, IRP, WF, CS	399.60	48	276.38 **	0.14	0.11	0.81	0.73
Model 5	PT + IRP, WF, CS, CE	417.55	48	294.33 **	0.14	0.12	0.80	0.72
Model 6	PT + WF, IRP, CS, CE,	378.45	48	255.24 **	0.14	0.08	0.82	0.75
Model 7	PT + CS, IRP, CE, WF	237.58	48	114.36 **	0.10	0.09	0.90	0.86
Model 8	PT, CE + WF, IRP, CS	221.49	48	98.27 **	0.10	0.90	0.87	0.07
Model 9	PT, CE + CS, WF, IRP	234.71	48	111.49 **	0.10	0.90	0.86	0.08
Model 11	PT, CE, WF + IRP, CS	372.22	48	249.00 **	0.14	0.82	0.75	0.10
Model 12	PT, CE, WF, IRP + CS	394.22	48	271.00 **	0.14	0.10	0.81	0.74
Model 13	PT + CE + WF + IRP + CS	712.92	54	589.70 **	0.18	0.12	0.64	0.56

Notes: PT = Perceived Task Demand, CE = Cognitive Engagement, IRP = In-Role Performance, WF = Work Fatigue, and CS = Cognitive Strain; ** *p* < 0.01.

**Table 2:** Mean, standard deviation, and correlations of variables.

Variable	1	2	3	4	5	6	7	8
1. Gender								
2. Education	0.09							
3. Tenure	0.05	−0.22						
4. Perceived Task Demand	0.03	0.03	0.03	(0.86)	0.47 **	0.43 **	0.36 **	0.15 **
5. Work Fatigue	−0.08	0.04	0.14	0.61 **	(0.83)	0.60 **	0.55 **	0.25 **
6. Cognitive Strain	0.08	−0.03	0.20	0.55 **	0.66 **	(0.70)	0.32 **	0.08
7. Cognitive Engagement	−0.13	0.08	0.01	0.46 **	0.59 **	0.31 **	(0.81)	0.46 **
8. In-Role Performance	−0.08	−0.09	0.13	0.24 *	0.34 **	0.16	0.60 **	(0.88)
*M*			29.08	3.07	3.22	3.10	3.59	3.70
*SD*			4.76	0.94	0.98	1.01	0.85	0.77

Notes: Correlations below the diagonal are at between-person level (i.e., for each employee, scores were averaged across all 5 days of the survey); correlations above the diagonal are at within-person level; ** *p* < 0.01 and * *p* < 0.05; values in parenthesis are Cronbach’s alpha values.

**Table 3:** Results of multilevel structural equation model.

Paths	Estimator	SE	95%LLCI	95%ULCI
Perceived Task Demand → Cognitive Engagement	0.13 **	0.04	0.05	0.21
Cognitive Engagement → In-Role Performance	0.35 **	0.03	0.05	0.21
Cognitive Strain → In-Role Performance	−0.17 **	0.03	0.29	0.41
Perceived Task Demand → In-Role Performance	−0.03	0.03	−0.09	0.03
Perceived Task Demand → Cognitive Strain	0.22 **	0.04	0.14	0.30
Cognitive Strain → Work Fatigue	0.10 **	0.04	0.02	0.18
Cognitive Engagement → Work Fatigue	0.01	0.04	−0.07	0.09
Perceived Task Demand → Work Fatigue	0.32 **	0.04	0.24	0.40

Note: ** *p* < 0.01.

**Table 4:** Results of Monte Carlo bootstrapping test.

Paths	Estimator	SE	95%LLCI	95%ULCI
**Direct Effect**				
Perceived Task Demand → In-Role Performance	−0.03	0.03	−0.09	0.03
Perceived Task Demand → Work Fatigue	0.32 **	0.04	0.24	0.40
**Indirect Effect (Within-Person Level)**				
Perceived Task Demand → Cognitive Engagement → In-Role Performance	0.05 **	0.01	0.02	0.08
Perceived Task Demand → Cognitive Strain → In-Role Performance	−0.04 *	0.01	−0.06	−0.02
Perceived Task Demand → Cognitive Engagement → Work Fatigue	0.01	0.01	−0.01	0.01
Perceived Task Demand → Cognitive Strain → Work Fatigue	−0.02 *	0.01	0.01	0.04

Note: ** *p* < 0.01; * *p* < 0.05.

**Figure A1:**
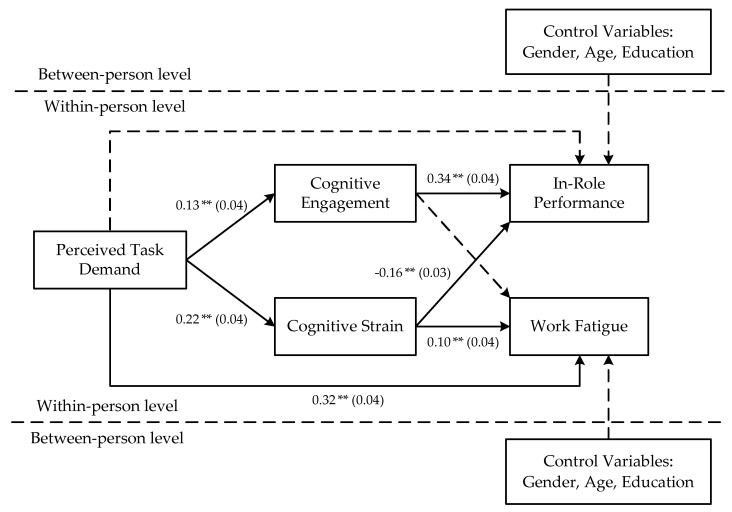
Conceptual model and results of the multilevel structural equation model with control variables. Notes: Values in parenthesis are standard errors; parameters are unstandardized; ** *p* < 0.01.

## Data Availability

The data presented in this study are available on request from the corresponding author. The data are not publicly available to honor respondents’ privacy.
